# A Test for Pus

**Published:** 1872-12

**Authors:** 


### ARTICLE XII.

### *A Test for Pus.*

Dr. Day, of Australia, has made some interesting observations on pus, which we quote from the London *Medical Times and Gazette:*

"In 1868," he observes, "I had the good fortune to discover a very delicate test for pus, and have since been in the almost daily habit of applying it, in conjunction with other tests, as aids to diagnosis. In this way I have learned some very interesting facts regarding the properties of pus. For instance, I have found that healthy pus, when dried, becomes chemically inactive; although, when moistened with water, it again resumes its chemical activity; also, that pus derived from persons suffering from diseases allied to erysipelas, possesses unusual activity—more than that from healthy persons—and which it is capable of retaining for years.

"On this paper are two spots of pus, which had been allowed to dry by exposure to the air. To one has been added the pus test alone, with, as you may see, a negative result, dry pus being devoid of chemical activity. To the other a drop of water is added, and then a drop or two of the pus test, with the result which always follows the application of this test to most pus—namely, a bright blue reaction.

"I mentioned just now that pus secreted by persons suffering from diseases allied to erysipelas is more active in its chemical properties than healthy pus. On this piece of glass is some pus taken from a large carbuncle on the neck of an elderly gentleman two years and three months ago. He was suffering from symptoms of blood poisoning at the time. This pus, as you will see, although it has been freely exposed to the air during the whole time, and sometimes to great heat, still retains its power of acting chemically on the pus test; and it does so even when dry, thus showing that it possesses greater chemical activity than ordinary pus.

"You will perceive that, in the explanation I have attempted regarding the influence of moist and dry air over the propagation of erysipelas and its allied diseases, I have assumed that when the chemical activity of pus is suspended, its power to act as a poison on the system is also suspended.

"I will trespass on your time by bringing one other experiment under your notice, as it may help to explain the *modus operandi* of Prof. Lister's antiseptic treatment of wounds.

"I have found that carbolic acid possesses the property of entirely and permanently destroying the chemical activity of pus, whether derived from healthy or unhealthy persons. On this paper is some pus which had been moistened with water, to give it chemical activity A few drops of watery solution of carbolic acid were then poured over it, and after the lapse of a quarter of an hour, the pus test was applied, with, as you may see, a perfectly negative result."

Dr. Day's pus test is so simple in the mode of appliance, and apparently so certain in its revelations, that we have little doubt it will soon come into daily use as an aid to diagnosis. He prepares his test fluid by exposing a saturated alcoholic solution of guaiacum to the air until it has absorbed a sufficient quantity of oxygen to give it the property of turning green when placed in contact with iodide of potassium. On moistening the most minute quantity of pus with water, and pouring a drop or two of the test fluid over it, a clear blue color is produced.—*Med. and Surg. Reporter.*

